# Dosimetric effects of internal margin, auto flash, and virtual bolus in VMAT‐based whole‐breast radiotherapy under motion variations

**DOI:** 10.1002/acm2.70471

**Published:** 2026-03-03

**Authors:** Ji Hyeon Joo, Dong Woon Kim, Wontaek Kim, Jiho Nam, Donghyun Kim, Dahl Park, Youn Joo Jung, Hyun Yul Kim, Ki Seok Choo, Kyung Jin Nam, Su Bong Nam, Jae Joon Kim, Yongkan Ki

**Affiliations:** ^1^ Department of Radiation Oncology Pusan National University School of Medicine Yangsan Republic of Korea; ^2^ Department of Radiation Oncology Pusan National University Yangsan Hospital Yangsan Republic of Korea; ^3^ Department of Radiation Oncology Pusan National University Hospital Busan Republic of Korea; ^4^ Department of Surgery Pusan National University Yangsan Hospital Yangsan Republic of Korea; ^5^ Department of Surgery Pusan National University School of Medicine Yangsan Republic of Korea; ^6^ Department of Radiology Pusan National University School of Medicine Pusan National University Yangsan Hospital Yangsan Republic of Korea; ^7^ Department of Plastic and Reconstructive Surgery Pusan National University School of Medicine Pusan National University Yangsan Hospital Yangsan Republic of Korea; ^8^ Department of Hematology and Oncology Pusan National University Yangsan Hospital Yangsan Republic of Korea

**Keywords:** breast neoplasms, intensity‐modulated radiotherapy, radiotherapy, surface‐guided radiotherapy, volumetric‐modulated arc therapy

## Abstract

**Background and purpose:**

This study aimed to evaluate the dosimetric performance and robustness of volumetric modulated arc therapy (VMAT) planning techniques—planning target volume with internal margin (INT), auto flash (AF), and virtual bolus (VB)—under simulated geometric changes during whole‐breast radiotherapy.

**Methods:**

Nine patients with left‐sided breast cancer were included. Three planning techniques were compared: INT with a 5‐mm skin‐sparing internal margin, AF with a 1‐cm automatic skin flash margin, and VB with a 5‐mm optimization bolus applied only during the planning. Respiratory motion was simulated by shifting the plan center (± 3 mm and ± 5 mm) and recalculating the dose distributions.

**Results:**

Under static conditions, all plans provided adequate target coverage, with planning target volume (PTV) V95% values of 96.45% (INT), 97.35% (AF), and 98.19% (VB). Under breast swelling of up to 5 mm, AF maintained the most stable coverage (PTV V95% = 99.10%), outperforming VB (95.02%) and INT (92.31%) (*p* < 0.001). In simulated incomplete inspiration (5 mm), VB showed superior robustness, achieving a PTV V95% of 90.46% compared with AF (85.30%) and INT (85.08%) (*p* = 0.008). AF met the ideal criteria in all cases under swelling conditions, whereas the VB was more effective against suboptimal respiration.

**Conclusions:**

In VMAT breast radiation therapy (RT), the conventional INT approach was the least robust against variations, and additional techniques are required. AF effectively compensates for breast swelling, whereas VB preserves the target coverage under insufficient breath‐hold conditions. Surface‐guided radiation therapy (SGRT) and visual guidance are recommended to ensure accurate treatment.

## 1 INTRODUCTION

Over the past two decades, the treatment paradigm for early stage breast cancer has shifted from radical mastectomy to breast‐conserving surgery (BCS) followed by adjuvant external beam radiation therapy (RT). This approach offers significant advantages over mastectomy, including reduced treatment‐related toxicity, and improved cosmetic outcomes and quality of life.^1^, ^2^, ^3^

Advancements in intensity‐modulated RT (IMRT) have further improved dose homogeneity and spared critical structures (the heart, lungs, and contralateral breast) during breast RT. Unlike conventional tangential fields that use manual field extensions to ensure an adequate skin dose, IMRT techniques must address shallow buildup regions near the skin surface. In clinical practice, a 5‐mm layer beneath the skin is often excluded from the planning target volume (PTV) to spare the skin, an approach adopted from skin‐sparing mastectomy data.^4^, ^5^, ^6^, ^7^ Alternatively, manual fluence extension beyond the skin surface (often called a “skin flash”) is used to account for soft‐tissue swelling or deformation, which is analogous to field expansion in tangential techniques.

In recent years, volumetric‐modulated arc therapy (VMAT) has been increasingly used for breast and chest wall treatments.^8^, ^9^, ^10^ However, because VMAT delivers continuously modulated beams, the implementation of a manual skin flash is not straight‐forward.^11^ Specialized planning strategies have been developed to address these concerns.

Auto flash (AF), available in Elekta's Monaco treatment planning system, automatically allows multileaf collimator (MLC) leaves to project outside the body contour for certain beam segments while keeping them closed for others.^12^, ^13^ A virtual bolus (VB) is another approach that artificially extends the target volume outwards by applying an optimized bolus during planning, which is removed for the final dose calculation.^14^ This forces the MLC leaves to remain away from the breast surface during optimization, thereby effectively creating a dynamic flash region.^15^ Although both AF and VB strategies are widely used, their impact on the potential tradeoffs in target coverage versus dose to critical structures have not been systematically evaluated.

Despite the use of respiratory control, residual intrafractional changes remain a clinical concern in VMAT‐based breast radiotherapy.^16^, ^17^ Building upon this limitation, our study uniquely compared the robustness of three widely used planning strategies—internal margin (INT), auto flash (AF), and virtual bolus (VB)—under bidirectional static isocenter shifts. To our knowledge, this is the first study to systematically evaluate these techniques using a uniform dataset and a modeled geometric displacement approach, providing practical guidance for optimizing superficial target coverage under variable respiratory conditions.

## 2 METHODS AND MATERIALS

### 2.1 Patient selection and imaging

This study was approved by the Institutional Review Board (IRB No. 55‐2025‐043). Nine patients with left‐sided breast cancer who underwent BCS were included in the study. The median age was 54 years (range, 42–74 years), and breast size varied among individuals. All patients underwent 3D computed tomography (CT) simulations (GE LightSpeed RT 16; GE Healthcare, Chicago, IL) for treatment planning. The clinical target volume (CTV) was delineated according to consensus guidelines, encompassing all visible glandular breast tissues on CT. The CTV was limited anteriorly to within 5 mm of the skin surface and posteriorly to the anterior face of the pectoralis and serratus anterior muscles, excluding the chest wall, ribcage, and lungs. No regional lymph nodes were included in the treatment volume. All patients were treated in the deep inspiratory breath‐holding (DIBH) position with prior training. The median anterior chest wall expansion at full inspiration was 7 mm (range: 6–8 mm).

### 2.2 Treatment planning

The PTV was generated by expanding the CTV by 5 mm in all directions. In most cases, the PTV extended slightly outside the patient's body contour (PTV_ext). All treatment plans were created using the Monaco treatment planning system (version 5.11, Elekta AB, Stockholm, Sweden) with 6 MV photon beams. Each plan consisted of two partial arcs that were optimized to cover the entire breast. The prescribed dose was 50 Gy, delivered in 25 fractions, with no additional boost dose. Three different planning techniques were generated for each patient:

1. PTV_int (INT): A PTV with a 5‐mm internal skin‐sparing margin was used (i.e. the 5‐mm layer beneath the skin was excluded from the PTV_ext). No AF or VB was used.
2. PTV_ext AF: The full PTV_ext was used, and a 1‐cm AF margin was applied according to the Monaco TPS guidelines. Dose optimization and normalization were based on the PTV_int.
3. PTV_ext VB: Full PTV_ext was used, and a 5‐mm VB was applied during planning (with the AF turned off). The bolus was removed for the final dose calculation, and the plan was normalized to cover the PTV_ext.

### 2.3 Respiratory movement

To evaluate the robustness of each plan under respiratory change, simulated setup errors were introduced, and the plans were recalculated after shifting the isocenter. Interfractional breast change has been reported to average between 4.8 and 5.53 mm, which can be reduced by respiratory coaching.^18^, ^19^ Chopra *et al.* observed an average anteroposterior displacement of 4.8 mm during deep breathing, while Wang *et al.* reported inter‐session skin position changes averaging 5.53 mm. Intrafractional motion is generally smaller, typically within 1–3 mm.^16^, ^20^ Based on these data, 3 and 5 mm static isocenter shifts were selected.

Two scenarios were simulated: (1) insufficient breath‐hold, modeled by +3 mm and +5 mm isocenter shifts in the left‐anterior direction to represent shallower inspiration than planned, and (2) excessive inspiration or breast swelling, modeled by −3 mm and −5 mm shifts in the right‐posterior direction to reflect chest over‐expansion. For each shift magnitude, the planned dose was recalculated on the original CT scan without re‐optimization to assess the changes in dose distribution and target coverage. In the VB plans, the bolus was removed before dose recalculation because no bolus was present during the actual treatment; therefore, the dose evaluation reflected the delivered scenario.

### 2.4 Statistical analysis

The primary dosimetric endpoints were PTV V95% (volume of PTV receiving ≥95% of the prescription dose), PTV D95% (dose covering 95% of the PTV), PTV Dmax (maximum point dose in the PTV), and dose heterogeneity index (HI). Organ‐at‐risk (OAR) metrics included the mean heart dose, ipsilateral lung V20 Gy and mean dose, and contralateral lung V5 Gy and mean dose. The differences among the three planning techniques were assessed using a one‐way analysis of variance. Post‐hoc pairwise comparisons were performed for endpoints that showed significant differences. Statistical significance was set at p < 0.05. All analyses were performed using R software (R Foundation for Statistical Computing, Vienna, Austria).

## 3 RESULTS

Table 1 summarizes the criteria used to evaluate the plan quality and the ideal, acceptable, and major deviation levels for each metric.

**TABLE 1:** Plan evaluation criteria for volumetric‐modulated arc therapy (VMAT) in whole‐breast radiotherapy.

Structures	Ideal	Acceptable variation	Major deviation
PTV V95%	≥95%	≥90%	<90%
PTV D95%	≥95%	≥90%	<90%
PTV Dmax	≤110%	<110%	>110%
Heterogeneity index	≤1.1	≤1.2	>1.2
Ipsilateral lung V20 Gy	≤15%	≤20%	>20%
Heart Dmean	≤5 Gy	≤6 Gy	>6 Gy

### 3.1 Dosimetric comparison of treatment plans

A summary of the target coverage and OAR doses for the three planning techniques (AF, VB, and INT) under static conditions is presented in Table 2. All three plans achieved adequate PTV coverage and dose homogeneity with no major violations of the planning criteria. The mean PTV V95% values were 97.35%, 98.19%, and 96.45% for AF, VB, and INT, respectively (*p* = 0.005). The mean PTV D95% values were 48.39 Gy (AF), 48.75 Gy (VB), and 47.95 Gy (INT) (*p* < 0.001), with VB plans achieving the highest minimum dose coverage. The maximum dose in the PTV (Dmax) averaged 53.67, 52.66, and 53.20 Gy for AF, VB, and INT, respectively (*p* = 0.012). There were no significant differences in dose homogeneity (HI) between the plans under static conditions (*p* = 0.273). Regarding OARs, the AF plans had a higher mean heart dose (7.56 Gy) than the VB (4.23 Gy) and INT plans (3.85 Gy) (*p* < 0.001). There were no significant differences in ipsilateral lung exposure, with V20Gy values of 12.77%, 13.06 %, and 12.56% for AF, VB, and INT, respectively (*p* = 0.836). The mean ipsilateral lung doses were 8.77, 9.07, and 8.75 Gy for AF, VB, and INT, respectively (*p* = 0.590). Contralateral lung exposure also showed no significant differences, with V5 Gy values of 7.95%, 16.22%, and 10.75% for AF, VB, and INT, respectively (*p* = 0.407). The mean dose to the contralateral lung was 2.34 Gy for AF, 2.59 Gy for VB, and 2.51 Gy for INT (*p* = 0.798).

**TABLE 2:** Comparison of dosimetric parameters among AF, VB, and INT plans under static conditions.

	AF (range)	VB (range)	INT (range)	*p*‐value
V95%	97.35 (96.18–99.45)	98.19 (97.02–99.58)	96.45 (94.96–98.2)	0.005
D95%	48.39 (47.997–49.268)	48.75 (48.3–49.267)	47.95 (47.492–48.439)	0.000
Dmax	53.67 (52.331–54.614)	52.66 (51.937–53.494)	53.2 (52.245–54.054)	0.012
D2%	51.76 (51.242–52.452)	51.44 (50.857–52.416)	51.51 (50.618–52.161)	0.347
HI	1.08 (1.04–1.25)	1.05 (1.04–1.07)	1.07 (1.05–1.08)	0.273
Ipsilateral lung				
V5 Gy (%)	52.56 (36.6–67.45)	57.15 (43.42–78.87)	53.87 (43.67–67.89)	0.487
V20 Gy (%)	12.77 (9.85–15.05)	13.06 (10.09–15.85)	12.56 (10.19–14.56)	0.836
D_mean_ (Gy)	8.77 (7.544–9.699)	9.07 (8.126–10.09)	8.75 (7.991–9.714)	0.590
Contralateral lung				
V5 Gy (%)	7.95 (0.44–34.66)	16.22 (1.09–50.15)	10.75 (0.15–36.45)	0.407
D_mean_ (Gy)	2.34 (1.784–4.445)	2.59 (1.844–4.689)	2.51 (1.664–4.626)	0.798
Heart				
D_mean_ (Gy)	7.56 (5.16–9.17)	4.23 (3.078–5.132)	3.85 (2.556–4.738)	<0.0001

Abbreviations: AF, Auto Flash; INT, Internal margin technique; VB, Virtual Bolus

### 3.2 Impact of geometric changes on dose coverage

**FIGURE 1:**
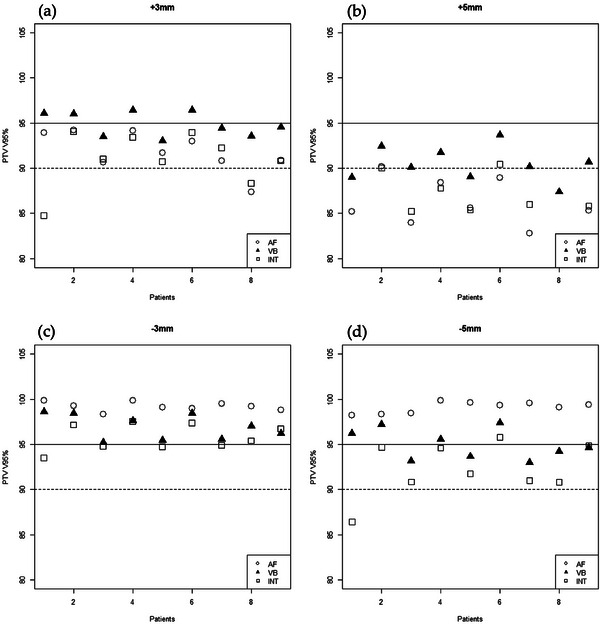
Individual patient PTV V95% under simulated setup shifts for each planning technique. Solid line: ideal threshold (≥ 95%); dotted line: acceptable threshold (≥ 90%). Panels represent different motion scenarios: (a) +3 mm, (b) +5 mm, (c) −3 mm, and (d) −5 mm.

The robustness of each plan under simulated geometric changes is presented in Table 3. For a + 3 mm incomplete inspiration (shallower breath‐hold), the VB plan maintained the highest PTV coverage (mean V95% 94.88%) compared with the AF (91.85%) and INT (91.04%) plans (*p* = 0.004), demonstrating superior preservation of dose coverage. In this scenario, two INT plans and one AF plan fell below the acceptable coverage levels (major deviations; Figure 1a). At a larger breath‐hold deficit of +5 mm, the target coverage decreased further in the AF (85.30%) and INT (85.08%) plans, whereas the VB plan achieved approximately 90.46% coverage (*p* = 0.008). In the +5 mm scenario, 6/9 VB, 0/9 AF, and 2/9 INT plans met the acceptable coverage criterion (PTV V95% ≥ 90%). However, the VB plans showed higher hotspot doses under these conditions: the average PTV Dmax increased to 56.11 Gy at +3 mm and 56.55 Gy at +5 mm, which were higher than those in the AF and INT plans (*p* < 0.001). At +3 mm, 7/9 VB plans, and at +5 mm, all VB plans exhibited a PTV Dmax above 110% of the prescription (major deviation), whereas no AF or INT plan exceeded this threshold. The HI remained similar among the three techniques for the +3 mm and +5 mm shifts (*p* = 0.144 and *p* = 0.401, respectively).

**TABLE 3:** Effect of simulated geometric changes on PTV coverage and dose metrics for INT, AF, and VB techniques.

	+3 mm				−3 mm			
	AF	VB	INT	*p*‐value	AF	VB	INT	*p*‐value
V95%	91.85	94.88	91.04	0.004	99.21	96.96	95.80	< 0.001
D95%	46.22	47.43	46.22	0.004	48.58	48.22	47.76	0.001
Dmax	53.32	56.11	53.49	< 0.001	56.21	55.21	53.74	0.003
D2%	51.54	53.94	51.56	< 0.001	53.26	53.08	51.58	0.001
V108%	0.00	2.64	0.00	0.006	1.54	0.40	0.01	0.013
HI	1.11	1.13	1.11	0.144	1.09	1.09	1.07	0.062

	+5 mm				−5 mm			
	AF	VB	INT	*p*‐value	AF	VB	INT	*p*‐value
V95%	85.30	90.46	85.08	0.008	99.10	95.02	92.31	< 0.001
D95%	43.33	45.08	43.51	0.025	48.34	47.54	46.62	< 0.001
Dmax	53.76	56.55	53.23	< 0.001	57.70	55.06	54.08	0.019
D2%	51.82	54.25	51.86	< 0.001	54.71	52.80	51.89	< 0.001
V108%	0.00	4.55	0.00	< 0.001	6.32	0.21	0.19	< 0.001
HI	1.26	1.19	1.18	0.401	1.11	1.10	1.10	0.649

**FIGURE 2:**
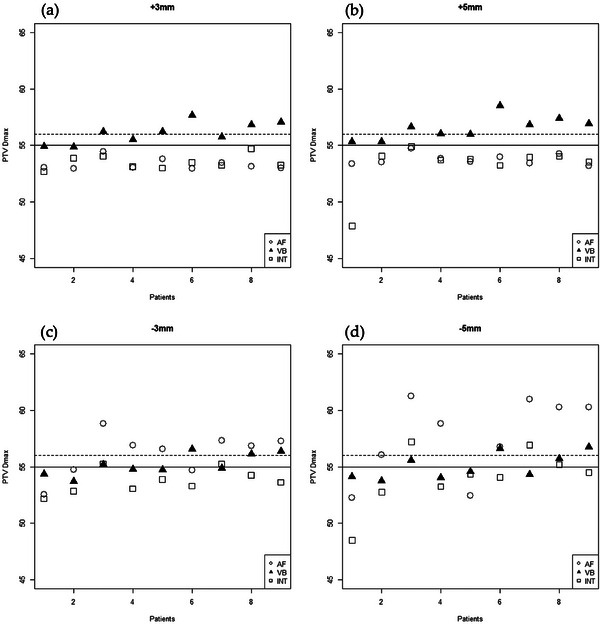
Maximum PTV dose (Dmax) distribution under simulated setup shifts. The Dmax values are shown for the AF, VB, and INT plans for each patient under the four‐shift scenarios. Doses exceeding 110% of the prescribed dose were considered major deviations.

Under the simulated breast swelling scenarios (negative shifts), all three techniques maintained excellent coverage for 3 mm swelling (−3 mm shift), with mean V95% values of 99.21% (AF), 96.96% (VB), and 95.80% (INT) (*p* < 0.001). Both the AF and VB plans met the ideal coverage criterion (V95% ≥ 95%) for all patients at −3 mm, whereas 4/9 INT plans dropped into the merely acceptable range (Figure 1c). At the extreme swelling of −5 mm, only the AF technique consistently maintained V95% ≥ 95% for all patients; the VB and INT plans showed reduced average coverage (95.02% and 92.31%, respectively; *p* < 0.001). As shown in Figure 2 (panels c and d), the AF plans incurred the highest Dmax under excessive swelling conditions, with six patients (at −3 mm) and seven patients (at −5 mm) experiencing a PTV Dmax of > 110%. No significant differences in HI were observed between the techniques for the −3 mm and −5 mm shifts (p = 0.062 and p = 0.649, respectively).

## 4 DISCUSSION

We assessed the dosimetric performance and robustness of the three VMAT planning techniques (INT, AF, and VB) under simulated geometric changes. The findings indicate that while INT plans remain a conventional approach for skin‐sparing, they exhibit the greatest susceptibility to changes, particularly under conditions of insufficient breath‐holding (+ 3 mm and + 5 mm errors). In contrast, VB demonstrated superior robustness in maintaining acceptable target coverage, whereas AF was the most effective in preserving target coverage during breast swelling of ≤ 5 mm. These results underscore the necessity of treatment planning strategies to ensure dosimetric stability during whole‐breast RT using VMAT.

Soft‐tissue deformations and setup variations are common in breast RT. Swelling of the breast tissue occurs in up to 50% of patients, typically ranging from 2 to 4 mm, with 13% exceeding 8 mm. In extreme cases, swelling can reach 15 mm, with 25% of patients experiencing deformations between 4 and 27 mm.^21^, ^22^, ^23^, ^24^ Given these potential changes, maintaining adequate PTV margins and using adaptive planning strategies are essential. Surface‐guided radiotherapy (SGRT) has been widely used to improve setup accuracy by monitoring a patient's surface in real‐time and reducing the reliance on skin tattoos. Studies have confirmed that SGRT improves setup consistency, particularly in terms of DIBH reproducibility.^25^ For example, Penninkhof *et al.* observed that SGRT reduced the mean breath‐hold positional variability from 2.10 mm to 1.69 mm (and further to 1.30 mm with visual feedback training). Additionally, implementing immediate corrections based on SGRT decreased the mean setup error from 3.9 mm (with no correction) to 1.4 mm.^26^ However, even with SGRT, interfractional setup shifts remain a challenge. Rudat *et al.* reported significantly larger day‐to‐day setup errors compared to intrafraction motion (*p* < 0.001), with mean residual shifts of approximately 1.9 mm laterally, 1.3 mm longitudinally, and 1.4 mm vertically—necessitating PTV margins on the order of 5–6 mm.^27^ Likewise, Mankinen *et al.* found that the SGRT‐alone setup often left a systematic error (particularly in the vertical direction, averaging −8.6 mm) that was only corrected after cone‐beam CT (CBCT) alignment; they recommended at least a 5‐mm CTV‐to‐PTV margin in 85% of cases, even when SGRT was used. Some individual cases with SGRT only had notable undercoverage, with a CTV V95% as low as 92% for VMAT.^28^ Our findings support this, as the INT plans exhibited the lowest robustness under respiratory and soft‐tissue deformation conditions. Therefore, alternative strategies such as AF and VB should be considered to enhance skin dose coverage and maintain dosimetric stability against interfractional variations.

The AF technique was designed to compensate for uncertainties in VMAT by dynamically extending the field as required. It introduces a controlled “air gap” beyond the skin, allowing the dose to be deposited even if the breast extends slightly beyond its planning position, while avoiding an excessive buildup of dose on the skin. Wang *et al.* demonstrated that under static conditions, there was a minimal dosimetric difference between plans with and without AF. However, with increasing respiratory motion, non‐AF plans suffered a significant drop in surface dose and target coverage, whereas AF plans maintained adequate coverage even at the largest motion amplitude. When simulating inspiratory motion by 5 mm amplitude, the PTV coverage dropped from 95.3% to 83.3% in non‐AF plans, whereas AF plans maintained stable coverage above 94%.^19^ This concurs with our findings that the AF plans provided reliable target coverage during simulated over‐inspiration scenarios (−3 mm and −5 mm shifts) that mimic breast swelling or deeper‐than‐normal inspiration. Furthermore, Mankinen *et al.* incorporated a 20‐mm flash margin using AF in their VMAT planning for breast cancer and confirmed that their VMAT plans remained robust against realistic interfraction changes in contrast to analyses that did not use any flash margins.^28^, ^29^, ^30^

The VB technique uses a different approach to ensure superficial coverage by effectively treating the expanded target volume during the treatment planning. By pushing the MLCs outwards via a temporary bolus, the VB ensured that the dose buildup region was extended beyond the skin surface. As a result, under moderate levels of tissue deformation, VB allows VMAT plans to retain their dosimetric advantages while achieving robustness comparable to conventional field‐in‐field techniques. In such cases, the use of CBCT‐based matching is recommended to ensure anatomical alignment.^23^ Tyran *et al.* reported that adding a VB to VMAT significantly improved target coverage and dose homogeneity, providing greater robustness against anatomical changes than plans without a bolus.^15^ In their study, when the patient's anatomy changed during treatment, the mean CTV V95% on repeat CT was 98.9% with the VB and 92.6% without the VB. Rossi *et al.* explored various bolus configurations and identified an optimal setup of a 5‐mm PTV extension combined with an 8‐mm optimization bolus, which yielded superior maintenance of the target and skin dose compared to no‐bolus plans.^31^

Comparisons of AF and VB under conditions of tissue deformation have been previously reported, with VB demonstrating superior robustness in maintaining target coverage during VMAT planning. In one such study, scenarios involving breast swelling up to 12 mm were examined. The results showed that an AF‐only plan exhibited the steepest decline in PTV V95%—dropping from 97% to 68%—while a VB‐only plan showed a more moderate reduction to 85%, and a combined AF+VB approach further limited the drop to 86%.^32^ These results suggest that VB is more advantageous for countering excessive breast expansion or swelling.

Collectively, our findings and prior research suggest that, given that interfractional variations persist despite SGRT improvements, a 4–6‐mm radial PTV margin remains necessary to account for setup and deformation uncertainties. The introduction of an internal PTV for dose buildup should be avoided, as it may lead to unintended dose heterogeneity and compromise treatment accuracy. Careful evaluation of skin dose buildup is essential when determining PTV margins, particularly in VMAT‐based breast radiotherapy. Our experimental design revealed that each of the planning strategies exhibits distinct robustness profiles when subjected to bidirectional isocenter shifts, which commonly occur as geometric variations in clinical practice. AF has effectively compensated for breast swelling of up to 5 mm, making it a viable option for managing interfractional anatomical changes. Conversely, VB has shown superior robustness against incomplete inspiration compared with other methods, ensuring stable target coverage even under suboptimal breath‐hold conditions. In clinical implementation, AF may serve as the default option for patients with reproducible DIBH, whereas VB can be prioritized when breath‐hold variability or shallow inspiration is anticipated. Further validation using motion‐tracked datasets will help confirm these strategy‐specific recommendations. This study has some limitations. Geometric changes were simulated using isocenter shifts rather than deformable dose recalculation on repeat imaging, which may not fully represent complex non‐rigid deformations. The sample size was small and limited to left‐sided DIBH cases, as all patients were analyzed using a standardized planning workflow with repeated recalculations to ensure consistency. While this design allowed meaningful statistical comparisons, extrapolation to larger deformations or different treatment setups should be made with caution. Nevertheless, the optimal approach is to maintain a consistent breath‐hold and ensure greater reproducibility of dose delivery. Further research should focus on the integration of AF and VB in VMAT planning, refining their application to maximize dosimetric accuracy while mitigating unnecessary dose buildup in the superficial tissues. Additionally, direct in vivo skin dose measurements and long‐term monitoring of skin toxicity are important for validating these strategies and ensuring their translation into clinical practice. In summary, this study is the first to directly compare the robustness of INT, AF, and VB strategies in VMAT‐based whole‐breast radiotherapy under bidirectional isocenter shifts. By linking specific change patterns to the optimal planning technique, our findings provide a practical, variation‐adapted framework for treatment planning. This represents the original contribution of our work to the field, extending beyond prior single‐technique evaluations and offering clinicians guidance for managing residual geometric changes in VMAT breast treatments.

## 5 CONCLUSIONS

This study demonstrated that AF effectively compensates for breast swelling up to 5 mm, whereas VB provides superior target coverage under incomplete breath‐hold conditions. The INT exhibited the lowest robustness to respiratory motion. A balanced approach incorporating AF or VB, along with optimized PTV margins is essential for maintaining dosimetric stability. Future research should investigate adaptive planning strategies and dynamic margin adjustments to enhance the robustness of treatment under variable breathing conditions. By addressing these challenges, this study establishes a foundation for more precise and personalized radiotherapy approaches, thereby im‐proving treatment effectiveness and patient safety.

## AUTHOR CONTRIBUTIONS

*Conceptualization*: Ji Hyeon Joo and Jae Joon Kim. *Formal analysis*: Jiho Nam. *Investigation*: Ki Seok Choo, Kyung Jin Nam and Su Bong Nam. *Methodology*: Youn Joo Jung and Hyun Yul Kim. *Software*: Dong Woon Kim and Dahl Park. *Validation*: Wontaek Kim and Donghyun Kim. *Writing—original draft*: Ji Hyeon Joo. *Writing—review and editing*: Yongkan Ki.

## ETHICAL APPROVAL

This study was approved by the Institutional Review Board of Pusan National University Yangsan Hospital (IRB No. 55‐2025‐043).

## CONFLICT OF INTEREST STATEMENT

The authors declare no conflicts of interest.

## Data Availability

The data that support the findings of this study are available from the corresponding author upon reasonable request.
